# Crucial Genes in Aortic Dissection Identified by Weighted Gene Coexpression Network Analysis

**DOI:** 10.1155/2022/7585149

**Published:** 2022-02-07

**Authors:** Hongliang Zhang, Tingting Chen, Yunyan Zhang, Jiangbo Lin, Wenjun Zhao, Yangyang Shi, Huichong Lau, Yang Zhang, Minjun Yang, Cheng Xu, Lijiang Tang, Baohui Xu, Jianjun Jiang, Xiaofeng Chen

**Affiliations:** ^1^Department of Cardiology, Taizhou Hospital Affiliated to Wenzhou Medical University, Linhai, 317000 Zhejiang Province, China; ^2^Department of Cardiology, Zhejiang Hospital, Hangzhou, 310013 Zhejiang Province, China; ^3^Department of Vascular Surgery, Taizhou Hospital Affiliated to Wenzhou Medical University, Linhai, 317000 Zhejiang Province, China; ^4^Department of Radiation Oncology, University of Arizona, Tucson, AZ 85721, USA; ^5^Department of Medicine, Crozer-Chester Medical Center, Upland, PA 19013, USA; ^6^Division of Vascular Surgery, Stanford University School of Medicine, Stanford 94305, USA; ^7^Department of Radiation Oncology, Indiana University School of Medicine, Indianapolis, IN 46202, USA

## Abstract

**Background:**

Aortic dissection (AD) is a lethal vascular disease with high mortality and morbidity. Though AD clinical pathology is well understood, its molecular mechanisms remain unclear. Specifically, gene expression profiling helps illustrate the potential mechanism of aortic dissection in terms of gene regulation and its modification by risk factors. This study was aimed at identifying the genes and molecular mechanisms in aortic dissection through bioinformatics analysis.

**Method:**

Nine patients with AD and 10 healthy controls were enrolled. The gene expression in peripheral mononuclear cells was profiled through next-generation RNA sequencing. Analyses including differential expressed gene (DEG) via DEGseq, weighted gene coexpression network (WGCNA), and VisANT were performed to identify crucial genes associated with AD. The Database for Annotation, Visualization, and Integrated Discovery (DAVID) was also utilized to analyze Gene Ontology (GO).

**Results:**

DEG analysis revealed that 1,113 genes were associated with AD. Of these, 812 genes were markedly reduced, whereas 301 genes were highly expressed, in AD patients. DEGs were rich in certain categories such as MHC class II receptor activity, MHC class II protein complex, and immune response genes. Gene coexpression networks via WGCNA identified 3 gene hub modules, with one positively and 2 negatively correlated with AD, respectively. Specifically, module 37 was the most strongly positively correlated with AD with a correlation coefficient of 0.72. Within module 37, five hub genes (AGFG1, MCEMP1, IRAK3, KCNE1, and CLEC4D) displayed high connectivity and may have clinical significance in the pathogenesis of AD.

**Conclusion:**

Our analysis provides the possible association of specific genes and gene modules for the involvement of the immune system in aortic dissection. AGFG1, MCEMP1, IRAK3, KCNE1, and CLEC4D in module M37 were highly connected and strongly linked with AD, suggesting that these genes may help understand the pathogenesis of aortic dissection.

## 1. Introduction

Aortic dissection (AD) is a lethal vascular disease characterized by the separation of intima from media and the formation of a false lumen, causing pulsatile blood flow into the aortic wall [1, 2]. In a large population study, annual AD incidence was 3.0 per 100,000 persons [3]. Currently, the exact mechanisms by which AD forms are not fully understood. A better understanding of gene expression in AD patients may help understand pathophysiology of AD and thus develop novel strategies for its diagnosis and treatment.

Bioinformatics is an effective tool for studying gene expression profiles and revealing potential molecular biological mechanisms. Genetic diseases often involve a network of different genes and pathways working together known as a gene module. Transcriptomics data from RNA sequencing (RNA-seq) was analyzed via coexpression network analysis to identify the functional association of genes with the disease [4]. The weighted gene coexpression network analysis (WGCNA) [5] constructs and analyzes gene modules coexpressing in a specific target group [6]. Differentially expressed gene (DEG) analysis and gene enrichment analysis have also been used to study gene expression in AD [7, 8] and found that some hub genes in AD were indicated [9, 10]. However, most studies used public databases such as Gene Expression Omnibus (GEO) database.

In this study, we analyzed genes in peripheral blood mononuclear cells (PBMCs) and the pathways associated with AD via high-throughput or next-generation sequencing (NGS) transcriptome. We constructed the gene expression modules using WGCNA and identified the hub genes in the related modules.

## 2. Materials and Methods

### 2.1. Human Clinical Specimens

In our study, 9 aortic dissection patients and 10 healthy controls in Taizhou Taizhou Hospital of Wenzhou Medical University (Zhejiang, China) were enrolled. Written informed consent was obtained from the patients or from their relatives. Peripheral blood samples were collected with the approval of the ethics committee of Taizhou Hospital of Wenzhou Medical University.

### 2.2. Next-Generation Sequencing Data Processing

Peripheral blood samples were collected into sterile EDTA-pretreated tubes, and PBMCs were isolated using the lymphocyte separation media. Total RNA was extracted using TRIzol™ Reagent (Invitrogen, Carlsbad, CA). RNA sequencing was performed using the BGISEQ-500 platform. The degree of gene expression was quantified by the number of uniquely mapped reads per kilobase of the exon region in a gene per million mappable reads (RPKM). R ×64 v. 3.5.1 (R Core Team 2018) and DEGseq are used for differential expression analysis with fold change ≥ 2 and adjusted *P* value ≤ 0.001.

### 2.3. Weighted Gene Coexpression Network Analysis (WGCNA)

A weighted gene coexpression network was constructed based on the genes of which FPKM > 0 across all samples with the help of R package WGCNA and build-in blockwise module function. First, a weighted adjacency matrix containing pair-wise connection strengths was constructed based on the selected soft threshold power (*β* = 10) on the matrix of pairwise correlation coefficients. This power adjacency implemented “soft” threshold to define connected neighbors of a gene that the network was weighted. Then, a topological overlap matrix (TOM) was generated from the adjacency matrix, and modules were defined as branches of a hierarchical clustering tree by using a dissimilarity measure, and each module was assigned a color. In our data, the minimum module size was set to 30 genes.

The first principal component of each module is denoted as module eigengene (ME), and modules with eigengenes more than 0.9 correlation would be remerged. The correlation between module eigengenes and the patients was calculated in order to find the modules that relate to AD. Correlation between gene expression values in each module and its module eigengene was also computed to measure the module membership (kME), the basis for hub gene selection. The connectivity of genes within each module was calculated (by softConnectivity function in WGCNA package), and the TOM of the top 200 genes was selected to visualize the module network. The top 500 of the resulting gene pairs ranked by weighted network edges were plotted using VisANT.

### 2.4. Gene Ontology and Pathway Enrichment Analysis

The Database for Annotation, Visualization, and Integrated Discovery (DAVID) was utilized to perform GO. For all analysis, the background was set to Homo sapiens (whole genome background), and the enrichment threshold was *P* < 0.05 (modified Fisher exact *P* value).

### 2.5. Statistical Analysis

Data are presented as the mean ± SEM. Statistical significance was determined with two-tailed *t*-test. The results were considered significant when *P* < 0.05. All gene set overlap was analyzed in MATLAB (MathWorks Inc., Natick, MA).

## 3. Results

### 3.1. Gene Expression Profile in AD Patients' PBMCs

**Figure 1:**
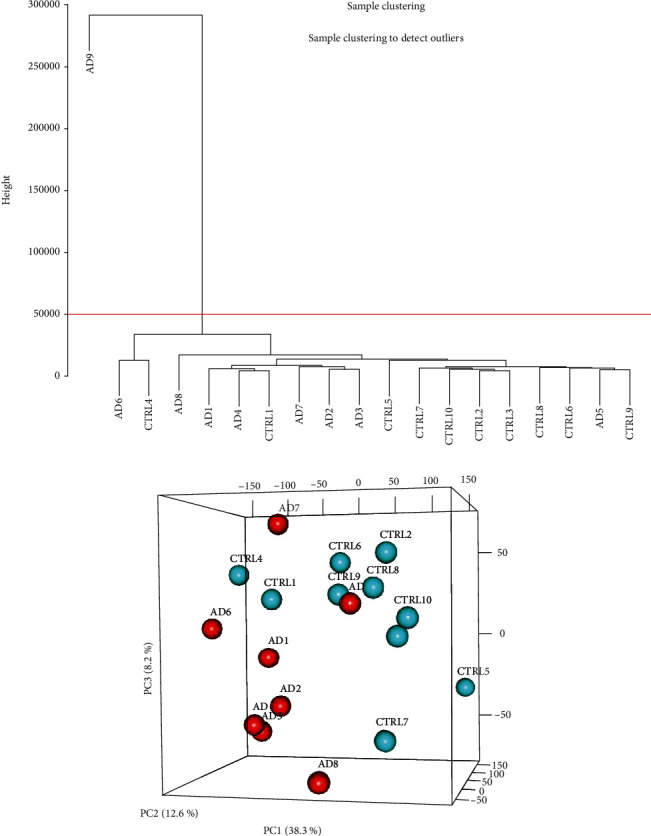
(a) Sample clustering. (b) 3D PCA plot.

Hierarchical clustering dendrogram was used to cluster genes based on gene FPKM profiles. Each sample represents a branch of the dendrogram. Gene expression of AD and health control patients was categorized into two distinct clusters: AD6 and CTLR4 as group one and the rest as group two, with the exception of the outlier of AD9 (Figure 1(a)). A similar result was attained by principal component analysis (PCA) (Figure 1(b)), confirming the differential expression of genes between AD and healthy control patients as two different gene clusters.

### 3.2. Identification of Differently Expressed Genes in AD Patients

**Figure 2:**
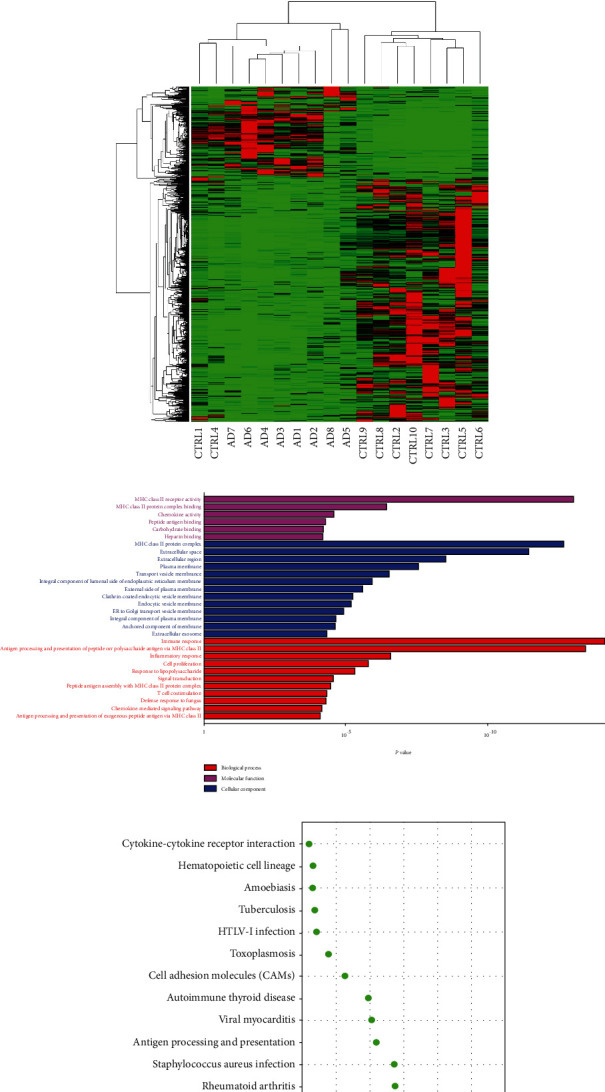
(a) Differentially expressed genes screened by DEGseq (fold change ≥ 2, *Q* values ≤ 0.001). (b) Heat map of differentially expressed genes (CTRL-vs-AD). (c) GO and (d) pathway enrichment analysis. ^∗^The size of the dot in (d) represents the number of genes counted within the pathway.

We identified a total of 18,817 genes. Using fold change ≥ 2 and adjusted *P* value ≤ 0.001 as threshold, a total of 1,113 genes were differently expressed between the healthy control and AD patients, with 812 genes downregulated and 301 genes upregulated in AD patients (Figures 2(a) and 2(b)).

GO analysis was conducted on DEGs to determine genes that were positively associated with AD in terms of molecular function (MF), cellular component (CC), and biological process (BP). Several important observations were found as shown in Figure 2(c): MHC class II receptor activity and MHC class II protein complex binding were the most significant enrichment in molecular function; MHC class II protein complex and extracellular space were the most notable enrichment in cellular component, and antigen processing and presentation of peptide or polysaccharide antigen via MHC class II and immune response were the most significantly positively enriched in biological process for the AD patients as compared to the healthy controls. As shown in the pathway enrichment analysis, the allograft rejection pathway was highlighted and also highly expressed in AD patients as compared to healthy controls (Figure 2(d)).

### 3.3. WGCNA Identifies Multiple Coexpression Module Markers Which Are Highly Related to AD

**Figure 3:**
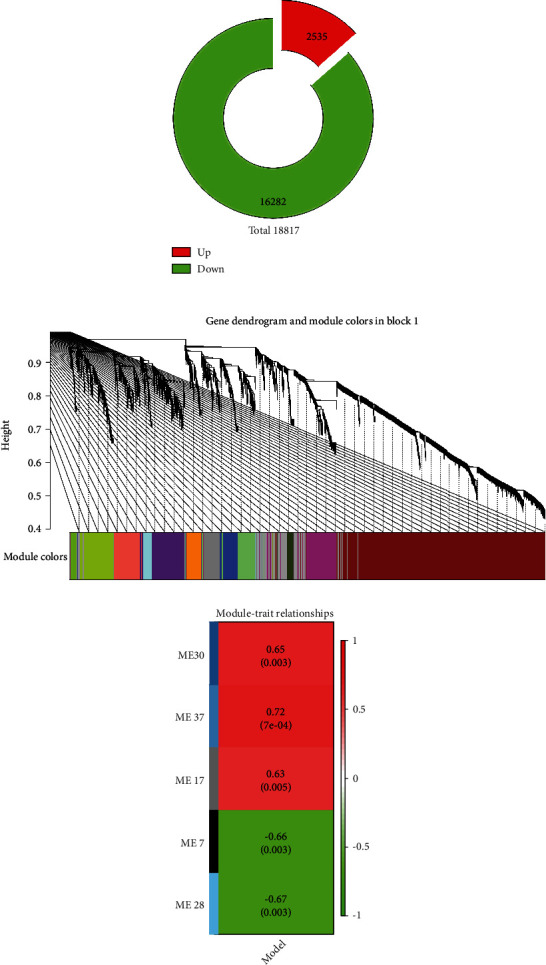
WGCNA by R. (a) Dataset used in WGCNA (FPKM > 0 across all samples). (b) Clustering dendrogram of genes in block 1, with dissimilarity based on topological overlap, together with assigned module colors. (c) Module-tissue (AD vs. CTRL) correlation and associated *P* values (in parentheses). Only shown modules with correlation above 0.6.

We constructed a weighted gene coexpression network based on genes which has FPKM greater than 0 across all samples (Figures 3(a) and 3(b)). Gene expression profiles were classified into 37 modules. The expression levels of each module were summarized by the first principle component (the module eigengene (ME)) and correlated with AD using Pearson correlation coefficient. In terms of correlation, M7(-), M17 (+), M28 (-), M30 (+), and M37 (+) were significantly associated with AD (∣correlation | ≥0.6, *P* < 0.05) (Figure 3(c)).

**Figure 4:**
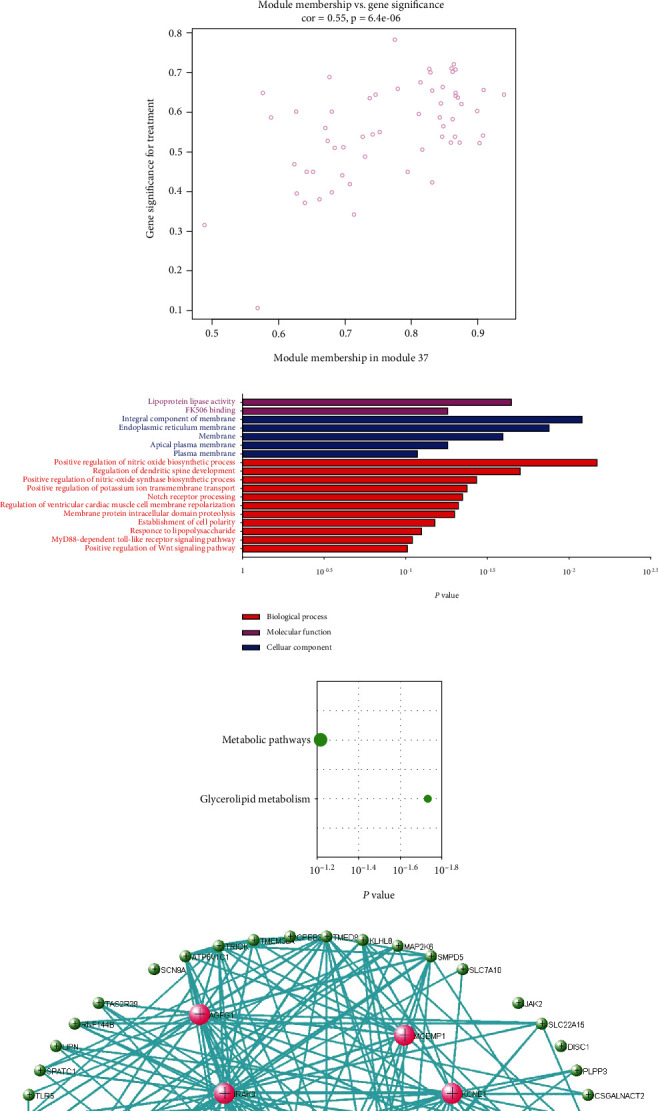
Analysis of M37. (a) Module heat map and the eigengene. (b) Scatterplot of gene significance (GS) for sample tissue vs. module membership (MM) in M37. There is a highly significant correlation between GS and MM in this module. (c) GO and (d) pathway of genes in M37. (e) Visualization of the network connections among the most connected genes in the M37 module, generated by the VisANT software.

Of the modules, M37 had the most significant positive correlation (*r* = +0.72) with AD (Figure 4(a)). This module demonstrated positive correlation between gene significance (GS) of sample tissues and module membership (MM) (Figure 4(b)). In Gene Ontology (GO) analysis, we identified 11 significant biological process (BP) terms (*P* < 0.05), most of which positively regulate nitric oxide biosynthetic processes. In terms of cellular component (CC) and molecular function (MF), integral components of membrane and lipoprotein lipase activity were enriched in AD patients (Figure 4(c)). Pathway analysis displayed significance of glycerolipid metabolism in AD patients as well (Figure 4(d)). The network connections among the most connected genes in the M37 module were AGFG1, MCEMP1, IRAK3, KCNE1, and CLEC4D, all positively correlated with AD patients (Figure 4(e)).

**Figure 5:**
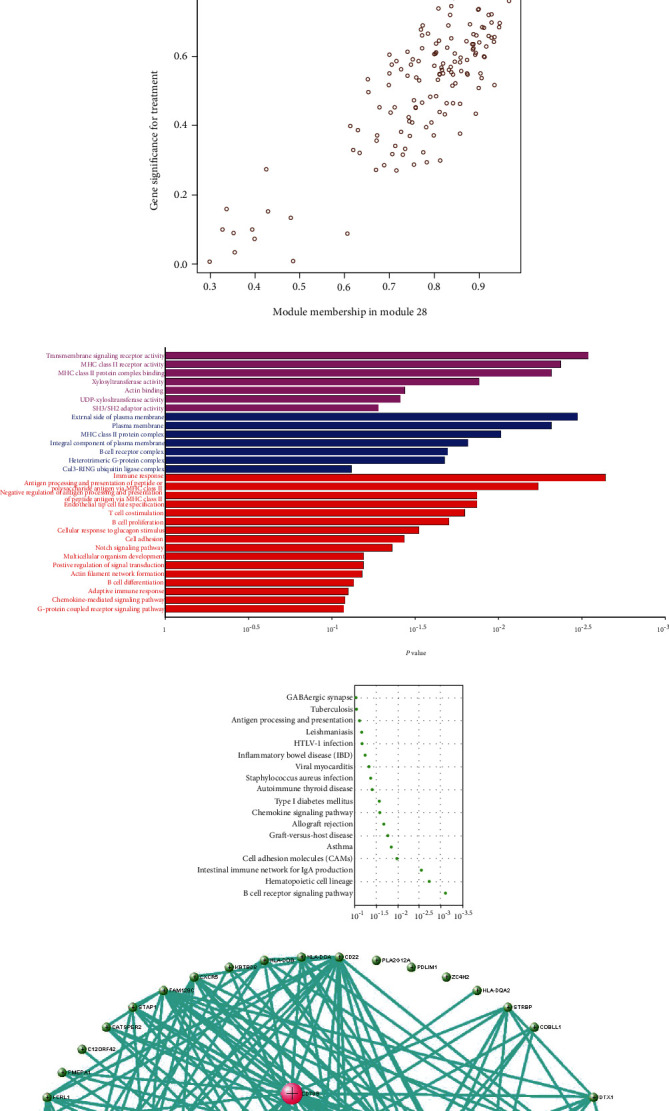
Analysis of M28. (a) Module heat map and the eigengene. (b) Scatterplot of gene significance (GS) for sample tissue vs. module membership (MM) in M28. There is a highly significant correlation between GS and MM in this module. (c) GO and (d) pathway of genes in M28. (e) Visualization of the network connections among the most connected genes in the M37 module, generated by the VisANT software.

M28 module was negatively correlated (*r* = −0.67) with AD (Figure 5(a)). However, when calculating the correlation between gene significance (GS) and each module membership (MM), the results demonstrated that M28 genes were also important elements of the MM (Figure 5(b)). Furthermore, GO analysis was performed for BP, MF, and CC terms. In the MF category, the DEGs were mainly involved in the transmembrane signaling receptor activity and MHC class II receptor activity. In the CC category, the DEGs were mainly related to the external side of the plasma membrane. In the BP category, the DEGs were mainly involved in immune response and antigen processing and presentation of peptide or polysaccharide antigen via MHC class II (Figure 5(c)).

Pathway analysis showed significance in B cell receptor signaling pathway for AD patients (Figure 5(d)). Five hub genes including CD79B, CD79A, PAX5, E2F5, and VPREB3 were obtained after the application of DEG for PPI network construction, and all had negative correlation with AD (Figure 5(e)).

**Figure 6:**
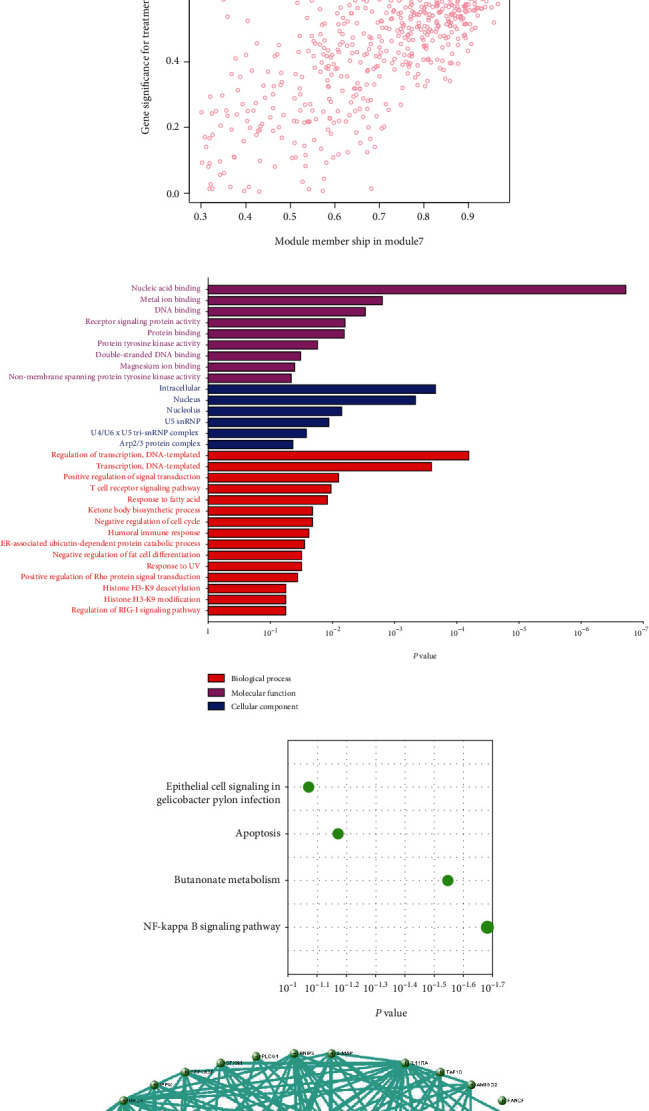
Analysis of M7. (a) Module heat map and the eigengene. (b) Scatterplot of gene significance (GS) for sample tissue vs. module membership (MM) in M7. There is a highly significant correlation between GS and MM in this module. (c) GO and (d) pathway of genes in M7. (e) Visualization of the network connections among the most connected genes in the M7 module, generated by the VisANT software.

M7 similar to M37 and M28 had significant correlation with AD with *r* =   − 0.66 (Figure 6(a)). GO analysis was performed to further analyze its function, and it displayed the significance of M7 in the NF-kappa B signaling pathway (Figure 6(c)). Genes in M7 were mainly involved in nucleic acid binding and regulation of DNA transcription (Figure 6(d)). The network connections among the most connected genes in the M7 module were generated. SNRPN, SNUPN, and MAGEH1 were identified to be negatively correlated to AD (Figure 6(e)).

## 4. Discussion

Aortic dissection is a severe, life-threatening disorder characterized by the tearing of the aortic wall. The compromised structural and functional properties of the aortic wall are recognized as the fundamental components of the underlying mechanism. Aortic wall inflammation, extracellular matrix degradation, smooth muscle cell dysfunction, and arterial wall remodeling are well recognized as the main mechanisms responsible for compromised aortic integrity [11, 12], with aortic wall inflammation being the most important contributing factor.

Various cardiovascular risk factors including age, hypertension, smoking, and atherosclerosis may increase the risk of AD. Apart from the acquired condition, inherent conditions like genetic disorders (Marfan syndrome, Loeys-Dietz syndrome, etc.) or tremendous nonsyndromic gene mutation (ATCA2, SMAD2, etc.) are associated with AD [13, 14]. Recently, there are emerging studies that revealed the interactions between some hub genes and AD, indicated some underlying pathways involving hub genes [9, 15], and utilized gene expression profiles to investigate the etiology and mechanism of AD [16, 17]. Our study utilized weighted gene coexpression analysis (WGCNA) to illustrate AD's underlying mechanism and uncover possible expression modules potentially correlated with aortic dissection.

We then performed functional analysis of GO enrichment and KEGG pathways on the DEGs (Figures 2(c) and 2(d)). Notably, GO analysis revealed that AD was positively correlated with the receptor activity, protein complex, protein complex binding, extracellular space, antigen processing, and presentation of peptide or polysaccharide antigen of MHC class II and immune response. Concerning KEGG pathways, DEGs were mostly enriched in the allograft rejection pathway. GO analysis and immune-related pathways were in accordance with previous research that innate and adaptive immune systems play an important role in AD's pathogenesis [18, 19].

WGCNA, constructed according to Pearson correlation, was used to identify gene coexpression networks related to AD clinical-pathological factors. We identified 37 distinct coexpression modules (denoted as M1 to M37). All 37 modules identified by WGCNA were correlated with aortic dissection. Through functional enrichment analysis, genes clustered in M37 have the highest positive correlation with AD. Further enrichment function analysis revealed the positive regulation of nitric oxide biosynthetic process and the glycerolipid metabolism pathway with AD. A PPI map was constructed in the M37, and top hub genes identified were AGFG1, MCEMP1, IRAK3, KCNE1, and CLEC4D which had positive correlation with AD.

In our study, AGFG1 (ArfGAP with FG repeats 1), including 17 mRNAs and 4 probable alternative promoters, has been reported to play a vital role in diseases, like HIV infection and asthma [20, 21]. Silencing of circAGFG1 significantly inhibited cell proliferation, migration, invasion, and stemness and promoted colorectal cancer cell apoptosis, tumor growth, and metastasis [22]. We speculate that the AGFG1 gene is associated with the expression of matrix metalloproteinases (MMPs) or underlying signal pathways which promote the recruitment of inflammatory cells, hence destruction of extracellular matrix and cell connections. Mast cell-expressed membrane protein 1(MCEMP1/C19OFR59) is a transmembrane protein found mainly on mast cells and macrophages that are involved in the pathogenesis of allergic and inflammatory diseases. Similar to many immune receptors, the gene promoter region contains nuclear factor kappa-light-chain enhancer of activated B cell and nuclear factor of activated T-cell binding motifs [23]. Previous research has highlighted the importance of MCEMP1 in sepsis, viral infection [24–26], and a potential useful prognostic tool in stroke patients [26]. Interleukin-1 receptor-associated kinase 3 (IRAK3) is a negative regulator for toll-like receptor (TLR) signaling pathways in innate and adaptive immune responses [27]. One study reported that IRAK3 deficiency exacerbates damages during ischemic events [28]. KCNE1 (minK) as a membrane-spanning protein modulates potassium channel functions in cardiac cellular electrophysiology, and its 112G > A polymorphism increases the risk of atrial fibrillation [29, 30] and is also associated with the long-QT syndrome and cardiac arrest [31]. CLEC4D (Dectin-2 cluster^″^ of C-type lectin receptor) is expressed exclusively by macrophages and is involved in the occurrence of pancreatic ductal adenocarcinoma [32]. A study by Kuo et al. demonstrated the hyperexpression of CLEC4D in peripheral leukocytes during acute phase of Kawasaki disease and its strong correlation to IVIG treatment resistance [33].

Current study suggests that the immune and inflammatory properties of several hub genes may be correlated with AD. Previous studies have deeply studied the relationship between AD and immune inflammatory mechanisms [34]. Kurihara et al. have demonstrated the correlation between AD and inflammatory response using animal models and found that matrix metalloproteinase and angiotensin II play significant inflammatory properties in AD [34]. Several research have further assessed the correlation between immune cells and hub genes through bioinformatics analysis based on GEO database. Chen and colleagues pointed out that several key immune cells such as macrophages, neutrophils, NKT cells, and natural Treg as well as inflammatory regulators including IL-6, CCL2, and HGF were involved in the development of TAAD [35]. Similarly, Gao and colleagues found that several hub genes including CA9, CXCL5, GDF15, and VEGFA were associated with monocyte or macrophage infiltration in dissecting aorta [36].

For the past decades, studies have revealed that aortic dissection-specific genes are inextricably linked with the abovementioned pathophysiological mechanisms; however, further studies are needed to explore the understanding of the disease. When evaluating the infiltration of immune cells in the aortic tissues using immunohistochemical methods, combination of the expression of the hub genes in tissue and in the circulation would be of great significance. Additionally, combination of our microarray data with the datasets downloaded from the GEO database would improve the quality of the research.

## 5. Conclusions

AD is a potentially lethal disease; therefore, prompt diagnosis and treatment are utmost important. Using weighted gene coexpression analysis, our study identified 5 key genes that were positively associated with AD. These genes play a vital role in immune regulation of the AD. The immune-related genes might help to shed light on the pathogenesis of AD and provide potential targets of prevention and immunotherapy for patients with TAAD.

## Data Availability

The datasets used and/or analyzed during the current study are available from the corresponding authors on reasonable request.
